# Relapsing TIPIC Syndrome after Administration of an mRNA-Based COVID-19 Vaccine

**DOI:** 10.1155/2023/6679200

**Published:** 2023-09-13

**Authors:** Georgiana C. Sandu, Gregor Weisser, Stefan Krämer, Matthias Reinhard

**Affiliations:** ^1^Department of Neurology and Clinical Neurophysiology, Germany; ^2^Center for Internal Medicine, Diabetology and Occupational Medicine, ZIDA Remstal, Germany; ^3^Department of Radiology and Nuclear Medicine, Medical Center Esslingen, Academic Teaching Hospital of the University of Tübingen, Germany

## Abstract

Reported vascular complications following mRNA-based COVID-19 vaccines are consisting of myocarditis, cerebral venous thrombosis, cerebral vascular thrombosis, and vaccine-induced thrombocytopenia. Here, we describe a case of a 49-year-old woman with left-sided pain above the middle common carotid artery (carotidynia) starting a few days after her second vaccination with an mRNA-based COVID-19 vaccine (Spikevax). Imaging was indicative of transient perivascular inflammation of the carotid artery (TIPIC) syndrome. The diagnostic workup for other immunologically mediated diseases was negative. The inflammation subsided after a course of prednisone and aspirin, and clinical symptoms vanished, but later mildly relapsed in the context of a viral upper respiratory tract infection other than SARS-CoV-2. Carotidynia because of TIPIC syndrome may present as an immunogenic side effect of the newly developed mRNA-based vaccinations against COVID-19. TIPIC syndrome should be considered in new-onset neck pain after vaccination.

## 1. Introduction

Perivascular transient inflammation of the carotid artery (TIPIC) syndrome, originally described by Temple Fay in 1927 as being characterized by tenderness and pain at the level of the carotid bifurcation (carotidynia) [1], is a rare encounter for the everyday practicing clinician. Five years ago, attempts were made to improve the diagnostic criteria for this yet unclassified clinic-radiologic entity. A trademark of this entity is a focal eccentric thickening of the carotid wall and abnormal soft tissue buildup surrounding the carotid artery in the setting of acute cervical pain, which may or may not be radiating to the head [2].

Multiple possible etiologies were reported over the years: postchemotherapy [3, 4], Burkitt's lymphoma [5], heralding the onset of acute leukemia [6], or fluoxetine-induced [7]. Here, we describe a case of TIPIC syndrome associated with an mRNA-based COVID-19 vaccination.

## 2. Case Presentation

A 49-year-old woman presented to our neurology department with acute left-sided ibuprofen-resistant neck pain overlying the left common carotid artery. The pain started 3 weeks ago with an exacerbation in the last 8 days without any associated focal neurological deficits. An ultrasonographic evaluation performed at an outpatient facility showed a wall thickening of the left CCA, hence the urgent referral to our clinic for further evaluation. Apart from hypertension and dyslipidemia with hypercholesterinemia, relevant medical history included a second mRNA-based COVID-19 vaccination with elasomeran (mRNA-1273, Spikevax®) in the left upper arm 7 days prior to symptom onset.

**Figure 1:**
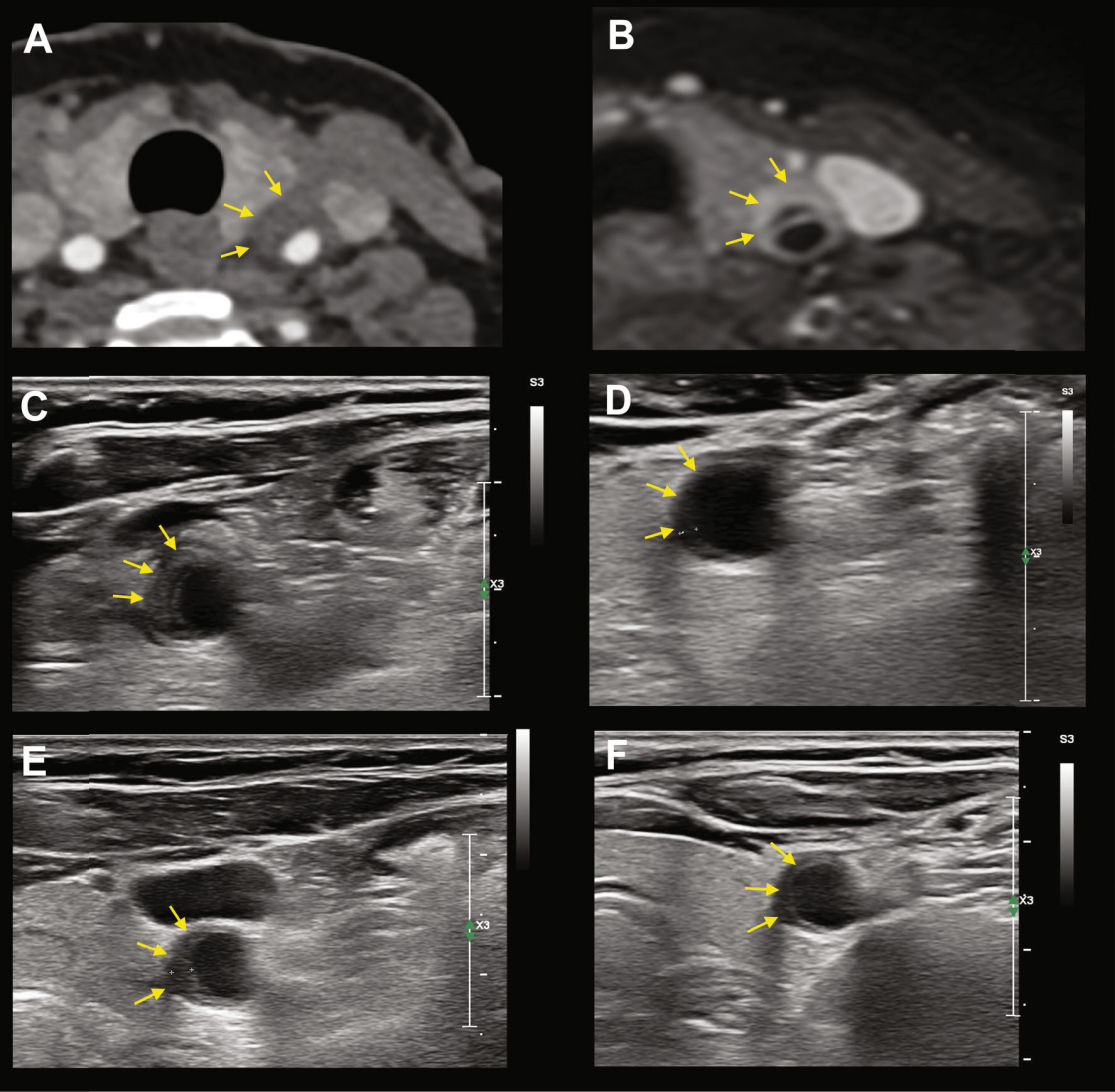
Imaging results. (a) The initial CT showing a perivascular soft tissue alteration due to inflammation and/or edema with slight luminal compression on axial slices. (b) MRI demonstrated a membranous structure within the CCA and a distinct ventrally accentuated circumjacent contrast enhancement on axial T1w fs images. (c) Initial ultrasound imaging with 18 MHz probe (Philips Epiq 5©). Axial imaging of the left common carotid artery in its middle segment, note the multilayered structure (arrows; maximum thickness 3.8 mm in axial plan, longitudinal extension 30 mm). (d) Follow-up imaging after 3 months showing regression of the eccentric vessel wall swelling (arrows; thickness in axial plan 1.1 mm). (e) Follow-up imaging after 6 months showing mildly relapsing wall swelling without a clear multilayered structure (arrows; thickness 2.5 mm). (f) Follow-up imaging after 9 months showing regression of the vessel wall thickening (thickness 1.4 mm).

CT angiography performed upon admission showed a semilunar hypodense thickening of the left CCA with a slight accompanying luminal narrowing. An additional MRI revealed a periarterial rather than ventrally situated STIR-signal enhancement in the middle third part of the left CCA on the postcontrast T1-weighted Dixon sequence. Furthermore, a membranous structure within the CCA and a distinct ventrally accentuated circumjacent contrast enhancement were found (shown in Figure 1). At this point, the possibility of a short CCA dissection was still being entertained. An ultrasonographic examination performed in our department revealed a focal eccentric 30 mm long medially located hypoechoic to moderately echogenic wall thickening of the middle third of the left CCA, which exhibited a multilayered appearance (shown in Figure 1(c)). A local, hemodynamically irrelevant stenosis of the left CCA was additionally seen. No intraluminal thrombus or dissection membrane could be demonstrated in the high-resolution sonographic exploration (18 MHz probe). A slightly elevated C-reactive protein (16 mg/l) could be seen on initial laboratory parameters. The vasculitis and sarcoidosis laboratory screenings were negative. The patient was given a short course of oral prednisone (40 mg, with a weekly tapering schedule of 5 mg) and Aspirin® due to the initially observed luminal stenosis. A repeat sonographic evaluation 3 days after treatment initiation showed slightly ameliorated findings, and the patient reported a significant pain improvement.

At follow-up examination after 3 months, a period in which the patient experienced a mild COVID-19 infection without any reoccurrence of the initial symptoms, revealed a marked improvement of the sonographic findings (shown in Figure 1). The patient reported complete recovery from neck pain. A repeated mRNA vaccination against SARS-CoV-2 was not performed.

A progression of the initially described lesions was found during the sonographic reevaluation after 6 months. The patient reported having experienced a 3-day moderate pain recurrence immediately before in the same location after sustaining an upper respiratory tract infection with a negative antigen test for SARS-CoV-2. With ibuprofen treatment (400 mg bid over 3 weeks), complete recovery of the pain occurred. An additional outpatient rheumatologic evaluation was unremarkable. At further follow-up 9 months after the initial presentation, the patient reported sustained recovery from pain over the left CCA. Left-sided subauricular pain and lymphadenopathy after a flu-like illness 14 days prior to presentation had been partially resolved under a short course of amoxicillin. On ultrasound, the eccentric wall swelling of the CCA had again regressed but was still moderately present, and the internal carotid artery was normal.

## 3. Discussion

TIPIC syndrome is a clinico-radiological entity characterized by unilateral or bilateral neck pain with tenderness superimposed over the carotid artery, intensified by movements such as swallowing, coughing, or sneezing. Its main complications are transient neurologic symptoms without underlying brain parenchymal lesions on subsequent MR imaging [2].

Pre-COVID literature reported TIPIC cases following flu-like illnesses or recent viral infections [1]. The outbreak of COVID-19 led to the recognition of a new potential etiology for this syndrome [8, 9] following COVID-19 infection. These cases reported unilateral neck pain onset after 2 weeks [8], respectively, or 1 month [9] after symptomatic SARS-CoV-2 infection, which then resolved either spontaneously [8] or after a course of levofloxacin and painkillers (unspecified) [9] within several days to a month. It has been demonstrated, meanwhile, also on engineered human blood vessel organoids in vitro [10], that SARS-CoV-2 can infect endothelial cells and promote inflammation using the angiotensin-converting enzyme 2 (ACE 2) receptor [11]. Immunohistochemistry and RT-PCR studies have confirmed SARS-CoV-2 direct invasion of the carotid body (CB), one of the structures with the highest blood flow (200 *mL* × 100 *g*^−1^ min^−1^), with subsequent histological alterations (inflammatory aggregates, microthrombosis, blood congestion, and microhaemorrhages) [12]. Consequently, TIPIC syndrome could be considered part of the intra- and perivascular inflammatory thrombotic manifestations due to COVID-19.

Only one other case of TIPIC syndrome after mRNA-based COVID-19 vaccination has been described recently [13]. In this case, a 39-year-old patient presented with ipsilateral neck pain, which debuted one week after the first vaccination with an mRNA-based Pfizer-BioNTech COVID-19 (BNT162b2) vaccine and later relapsed after a second vaccination with the same substance. A TIPIC syndrome diagnosis was made along with the recognition of supraclavicular lymphadenopathy on the same side. The patient received a 5-day course of a nonsteroidal anti-inflammatory drug (dexketoprofen, 50 mg daily) and reported complete symptom relief within 10 days. When the relapse occurred, he took the same medication, and he experienced symptom relief within 14 days. A control sonographic evaluation at 1.5 months revealed complete resolution of the focal eccentric thickening of the carotid wall.

Our present case is in accordance with the four major diagnostic criteria of TIPIC syndrome proposed by Lecler et al. [2]: (1) the presence of acute pain overlying the carotid artery, which may or may not radiate to the head; (2) eccentric perivascular inflammation on imaging; (3) exclusion of another vascular or nonvascular diagnosis with imaging; and (4) improvement within 14 days, either spontaneously or with anti-inflammatory treatment. Immediate ultrasonographic evaluation of the periadventitial changes underlying the painful area represents an easily available diagnostic tool, provided that the clinician is fully aware of the sonographic findings associated with this syndrome [14].

A relevant disadvantage of mRNA-based COVID-19 vaccines is that they seem to cause adverse reactions in those susceptible to an autoimmune response [15]. The time delay between vaccination and the onset of symptoms supports an immunogenic triggering postvaccination. Molecular mimicry, the production of particular autoantibodies, and the role of certain vaccine adjuvants were postulated as substantial contributors to autoimmune phenomena [15]; however, a specific immunologic pattern or antibody response has not been reported so far in patients with TIPIC syndrome.

Interestingly, a subsequent COVID infection did not lead to a relapse of symptoms in our patient, probably because of ongoing steroid treatment at this point. After complete withdrawal of steroids, however, the patient developed a relapse after an upper respiratory tract viral infection other than COVID-19. Relapsing TIPIC syndrome occurs in 19% of patients [2], 78% of whom exhibit a clinical history of autoimmune disease and initially present with a simultaneous acute exacerbation of their condition [2]. The observed reoccurrence in our patient suggests that more complex immunological mechanisms with a specific vulnerability may be at play. Further evaluation in a rheumatology clinic and the search for a potential autoimmune/inflammatory disease did not, however, reveal any abnormalities.

A prevalence of 2.8% of TIPIC syndrome among patients with acute neck pain has been described by Lecler et al. [2]. Being characterized by relatively minor and rapidly improving symptoms, the actual prevalence of this syndrome might be underestimated. Although the prevalence of neck pain after COVID-19 vaccination has not been formally investigated, in clinical practice, patients do report neck soreness or stiffness as a postvaccination side effect. Without any further investigation, the prevalence of TIPIC syndrome after vaccination might be underestimated.

In conclusion, TIPIC syndrome should be considered a potential differential diagnosis in acute neck pain following COVID-19 vaccination, which subsequently should prompt immediate ultrasonographic evaluation to reveal the periadventitial changes underlying the painful area.

## Data Availability

All data generated or analysed during this study are included in this article. Further enquiries can be directed to the corresponding author.
